# Supplementary material for: Human access impacts biodiversity of microscopic animals in sandy beaches
Source: Commun Biol. 2020 Apr 20;3:175. doi: 10.1038/s42003-020-0912-6 (PMC7170908; doi:10.1038/s42003-020-0912-6)
Supplement: Supplementary file 1 — Supplementary Information [file 42003_2020_912_MOESM1_ESM.pdf]

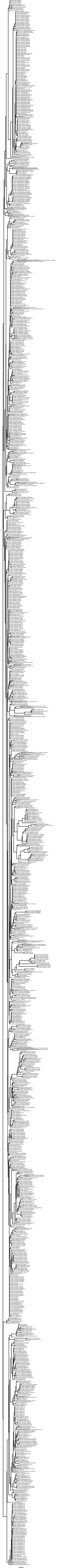

Supplementary Figure 1

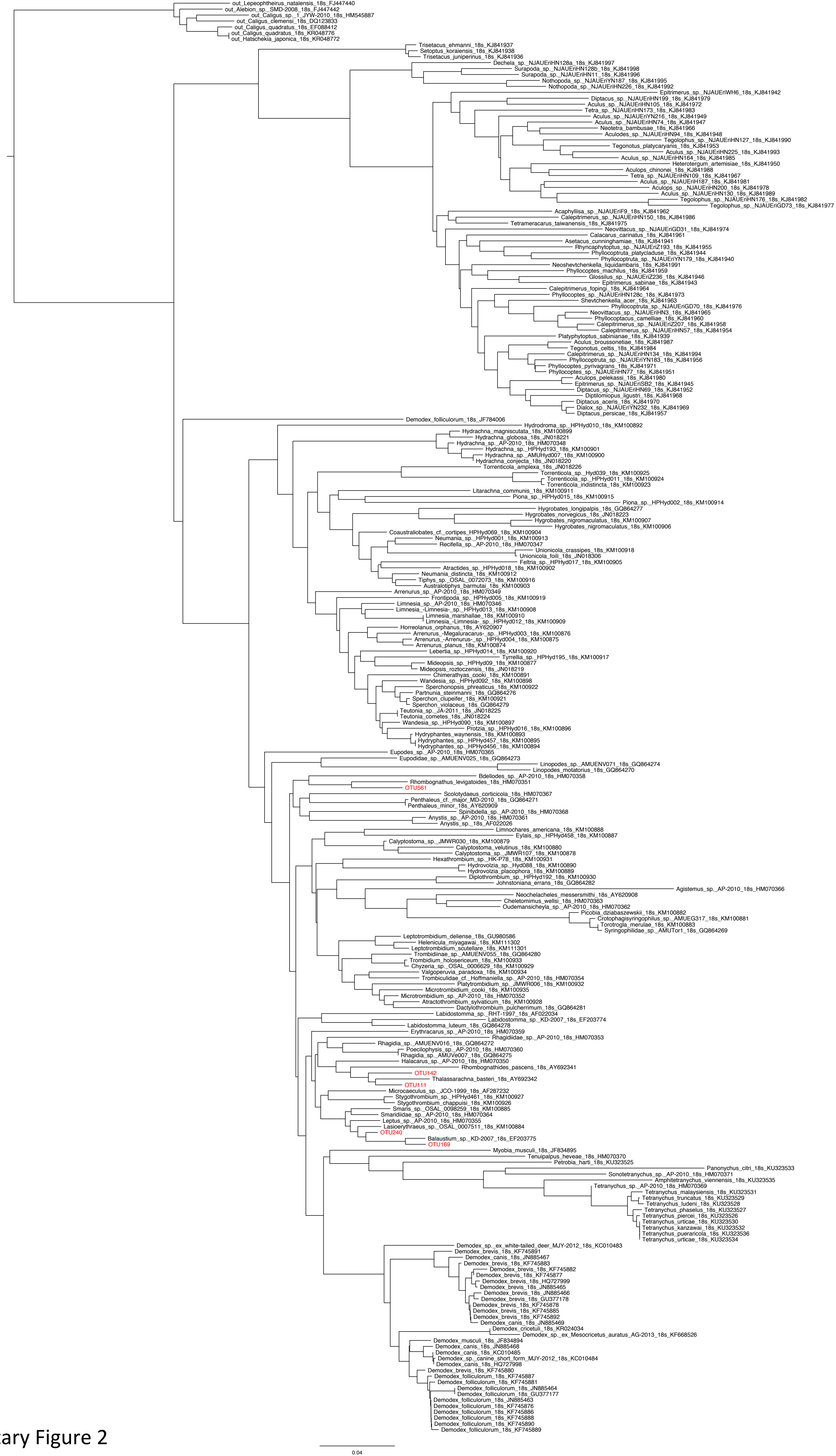

Supplementary Figure 2

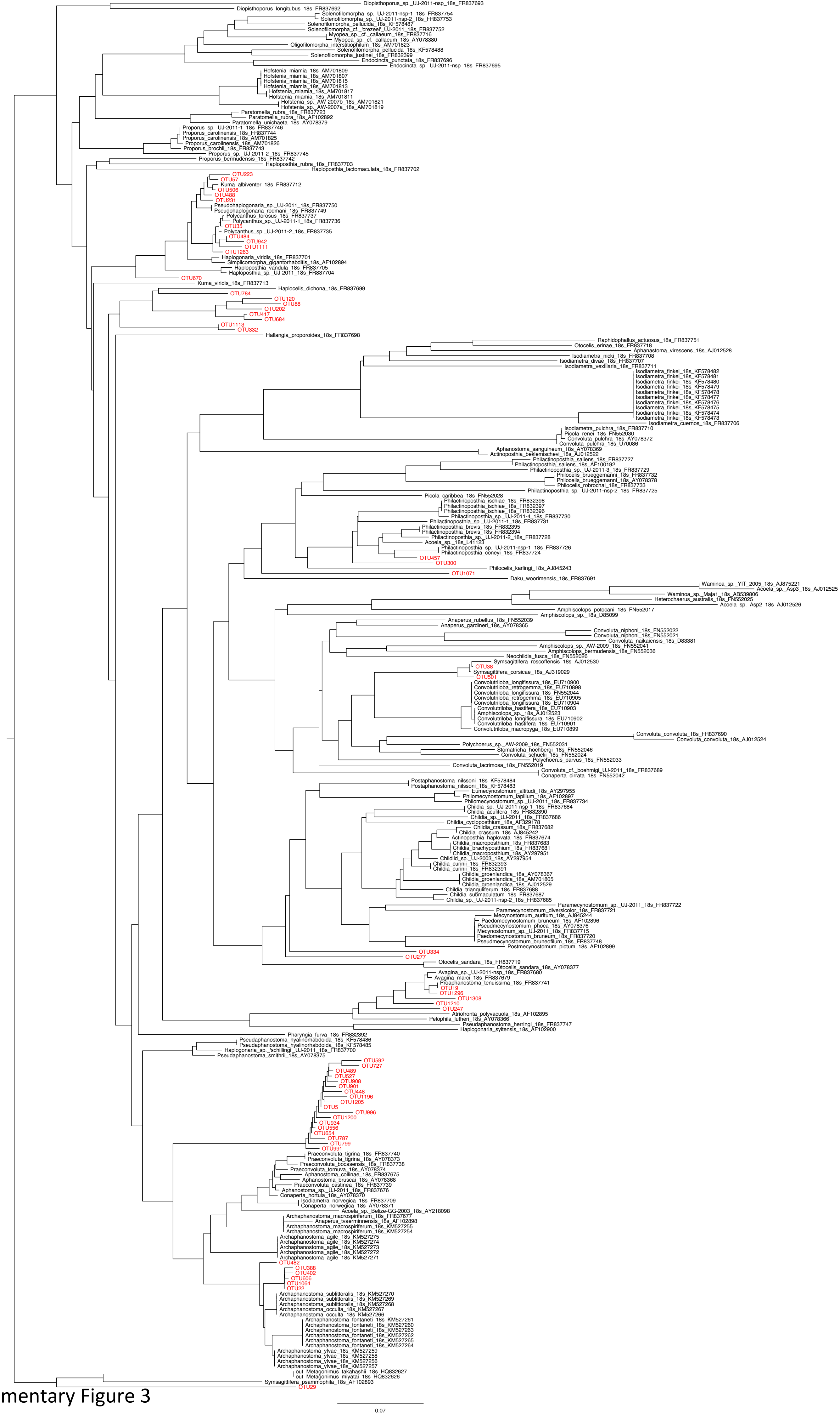

Supplementary Figure 3

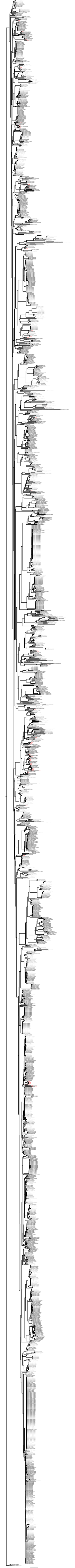

Supplementary Figure 4

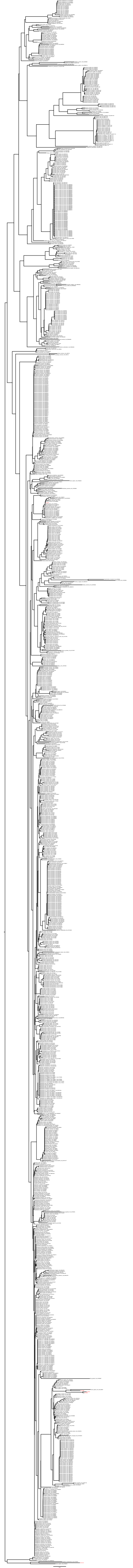

Supplementary Figure 5

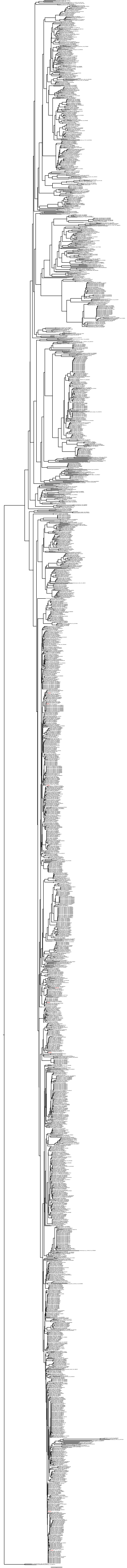

Supplementary Figure 6

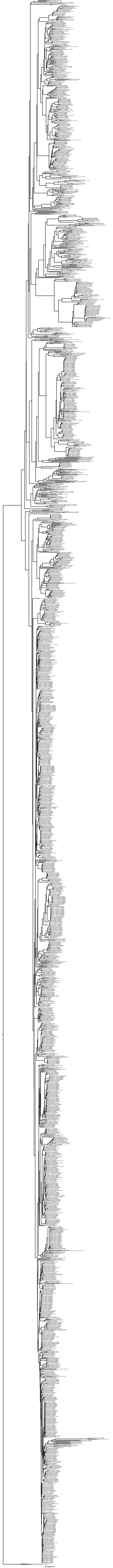

Supplementary Figure 7

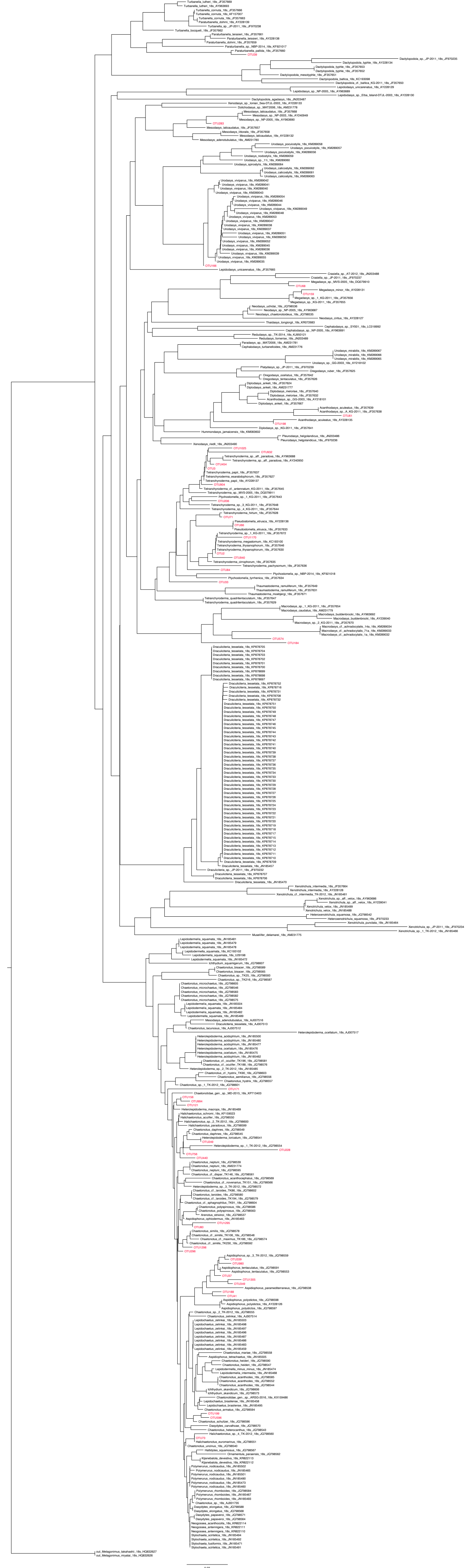

Supplementary Figure 8

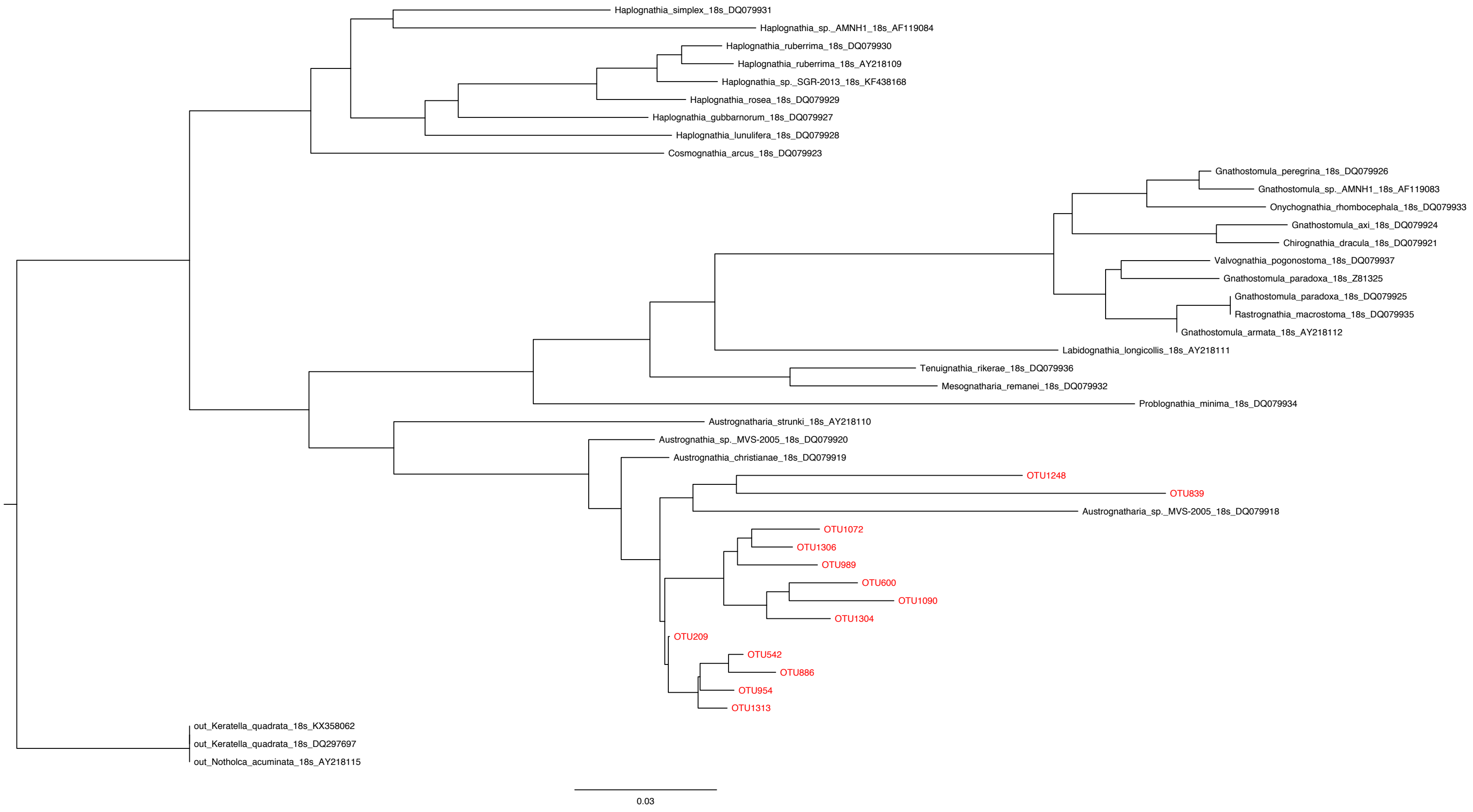

Supplementary Figure 9

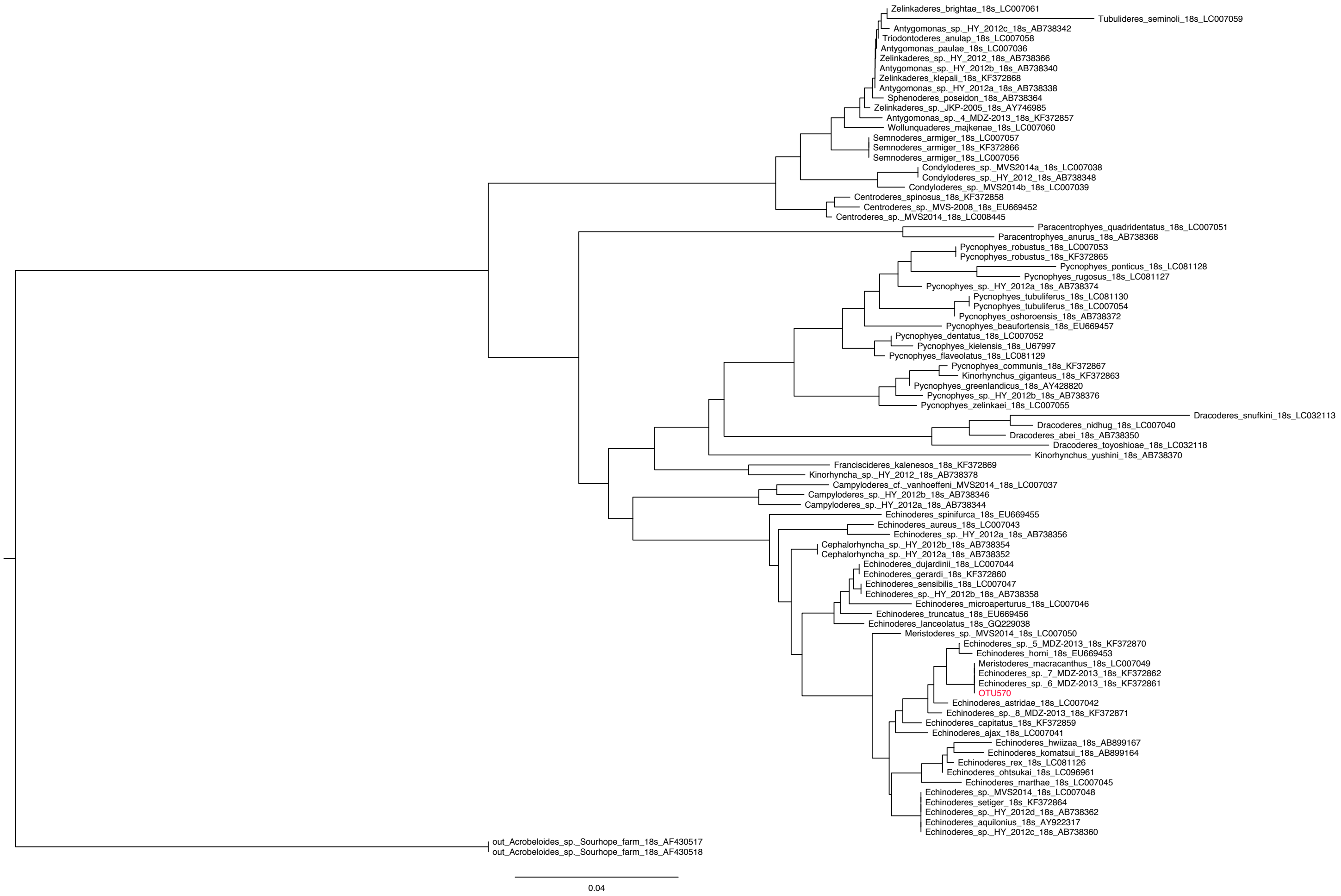

Supplementary Figure 10

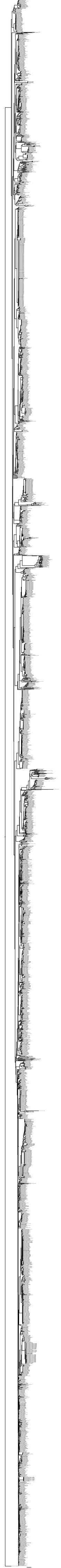

Supplementary Figure 11

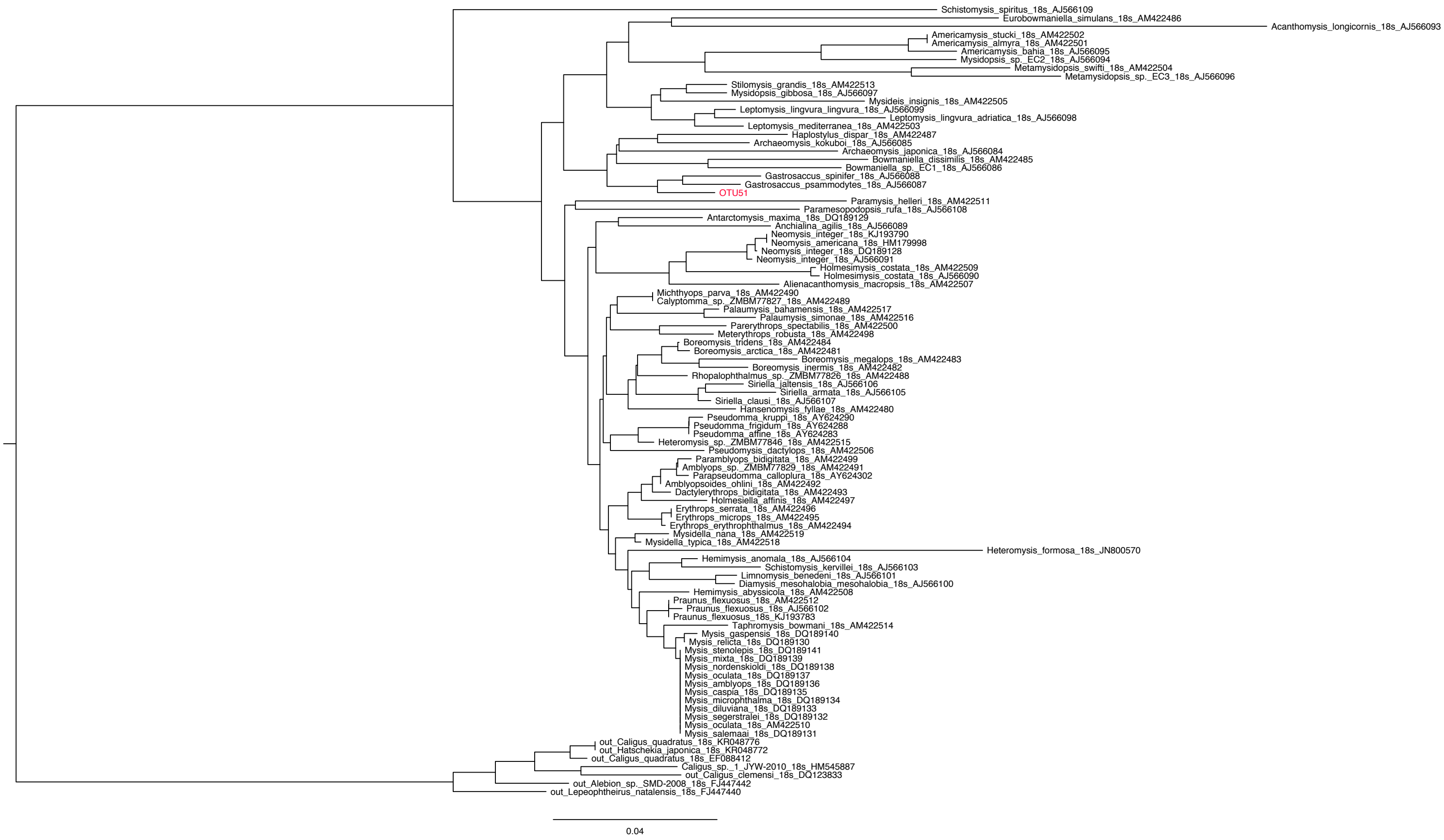

Supplementary Figure 12

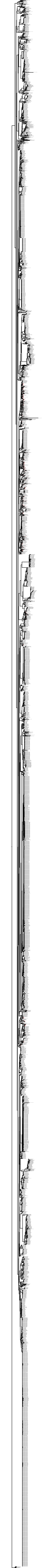

Supplementary Figure 13

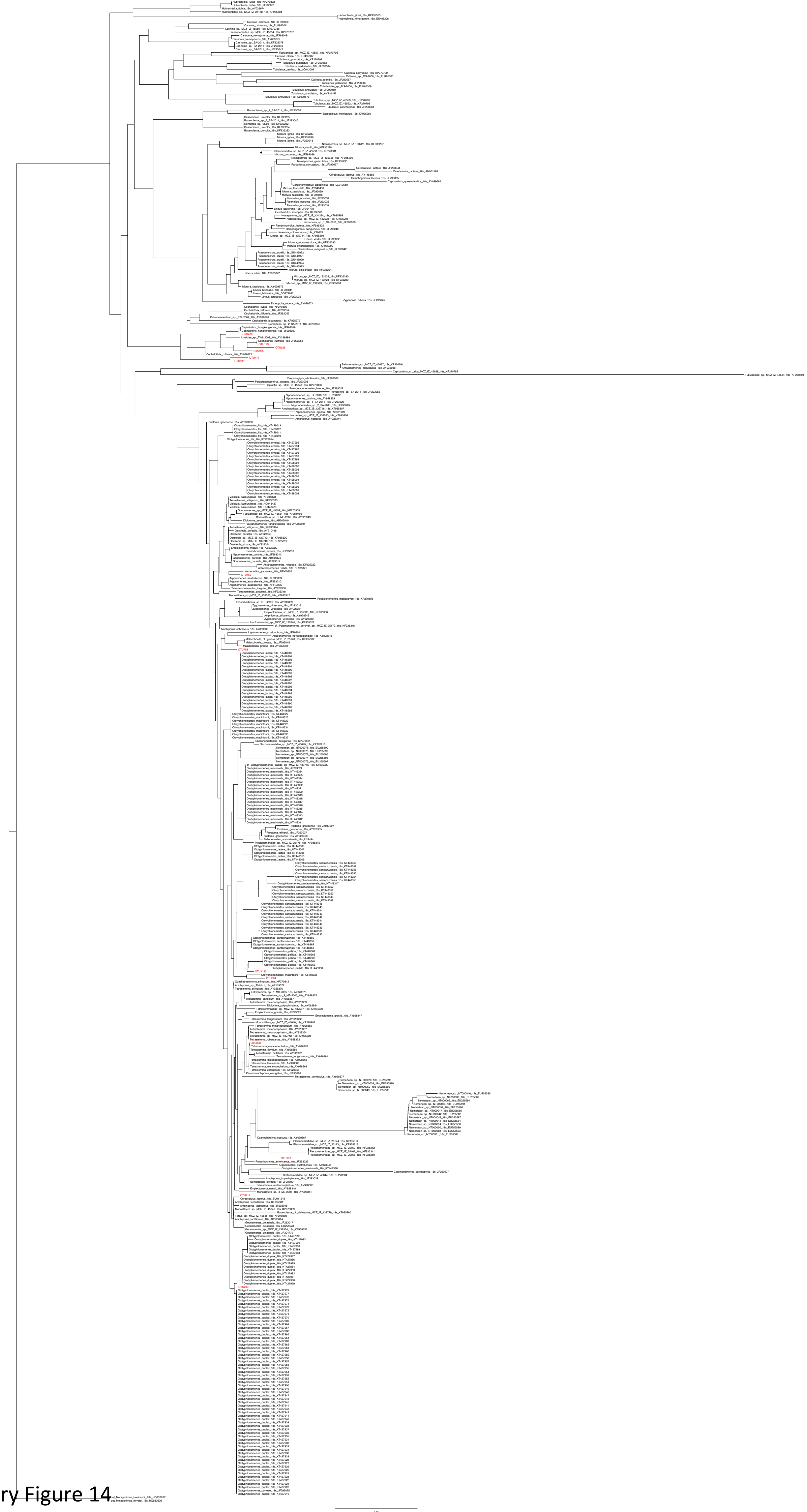

Supplementary Figure 14

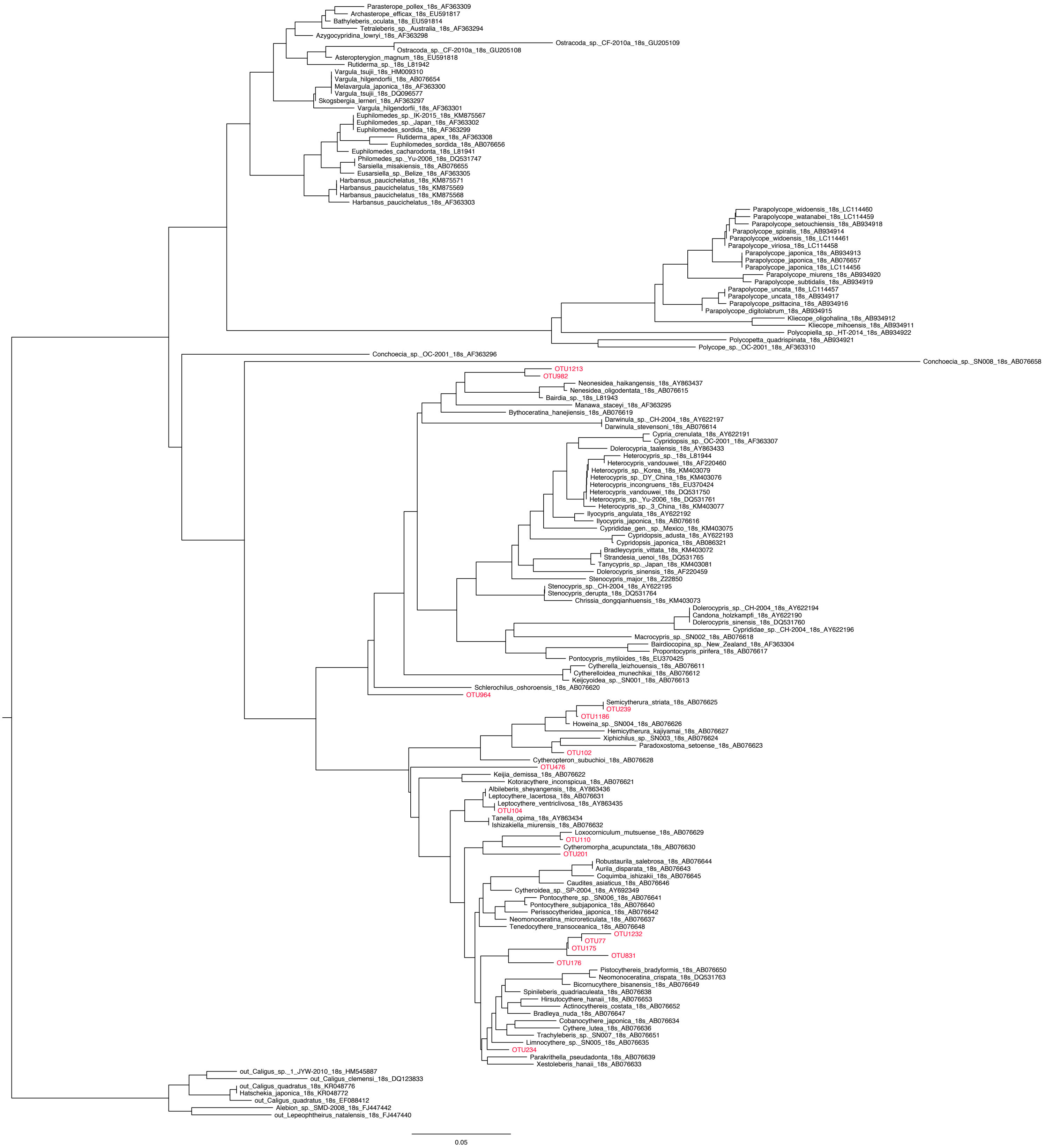

Supplementary Figure 15

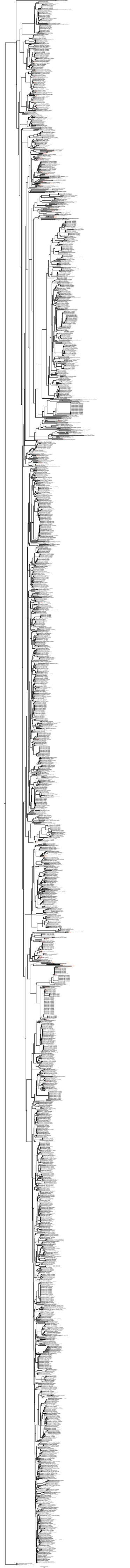

Supplementary Figure 16

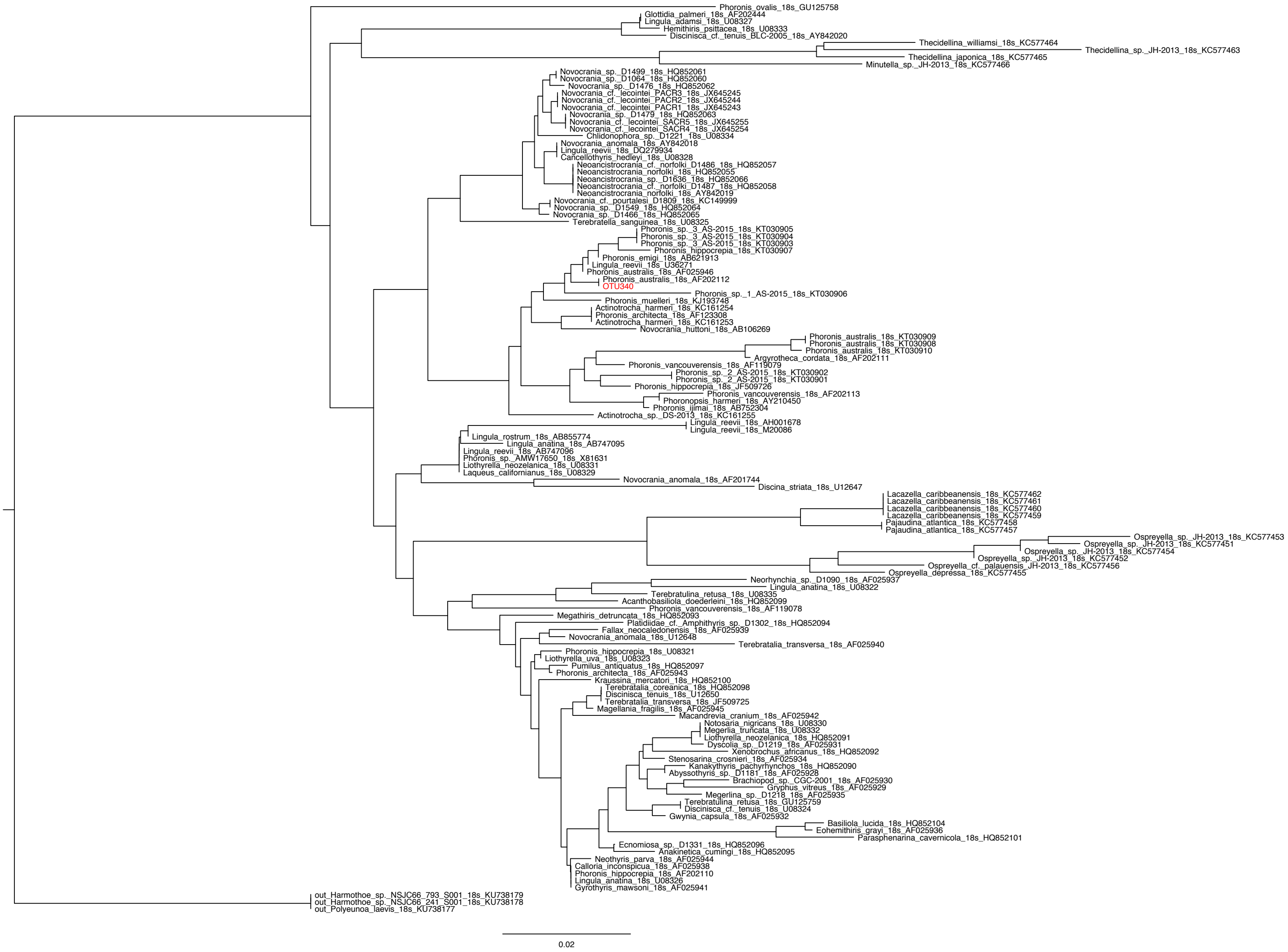

Supplementary Figure 17

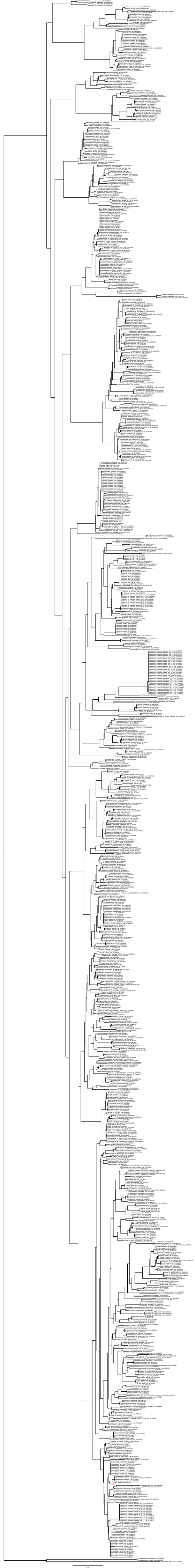

Supplementary Figure 18

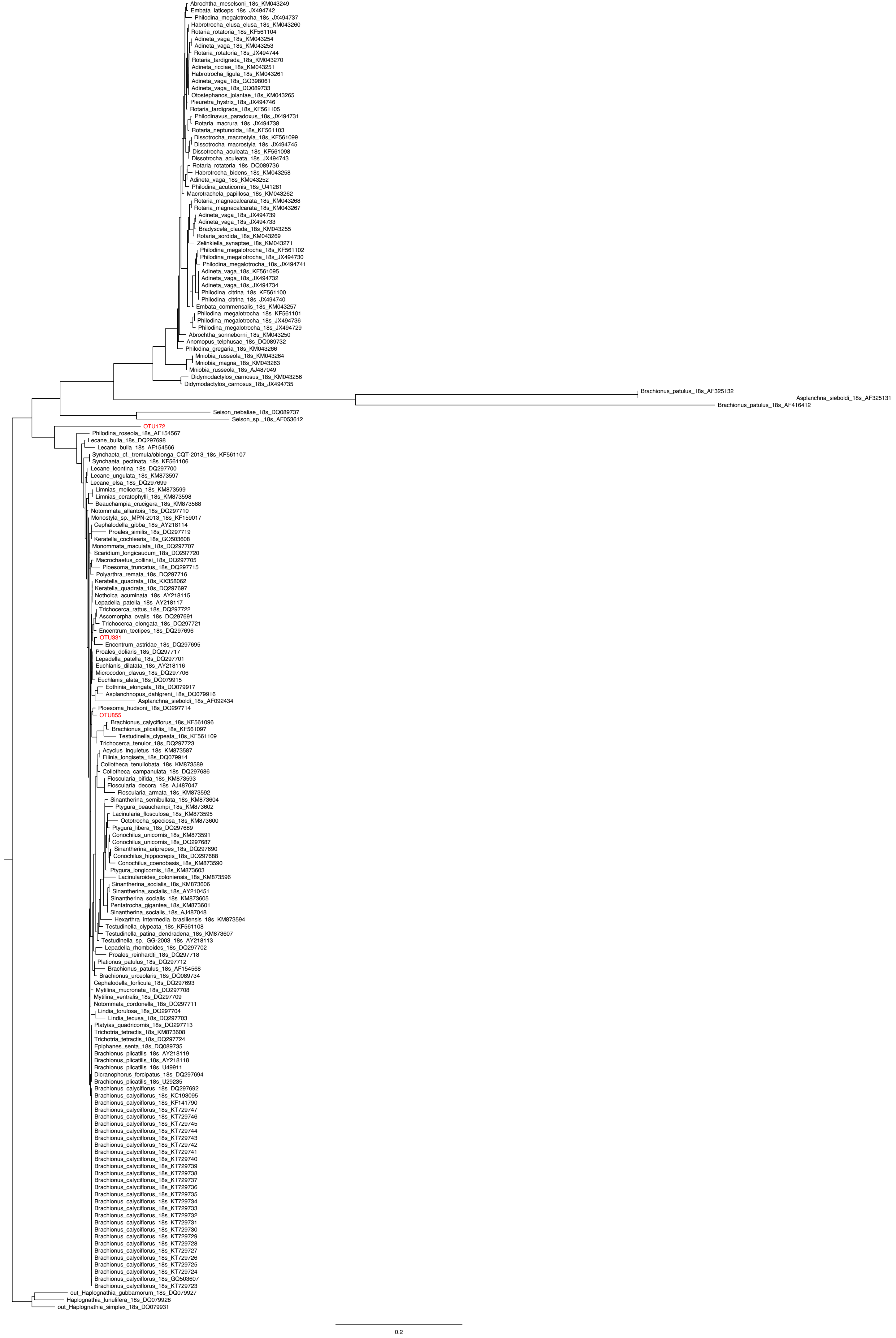

Supplementary Figure 19

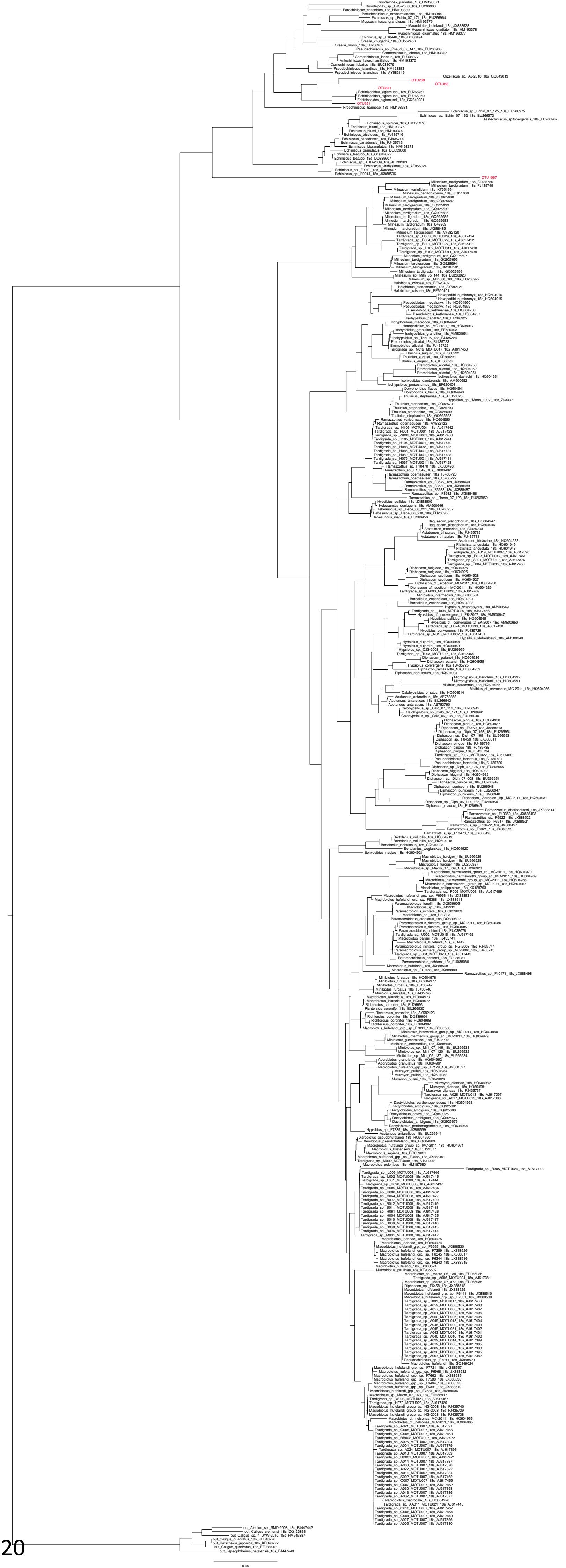

Supplementary Figure 20

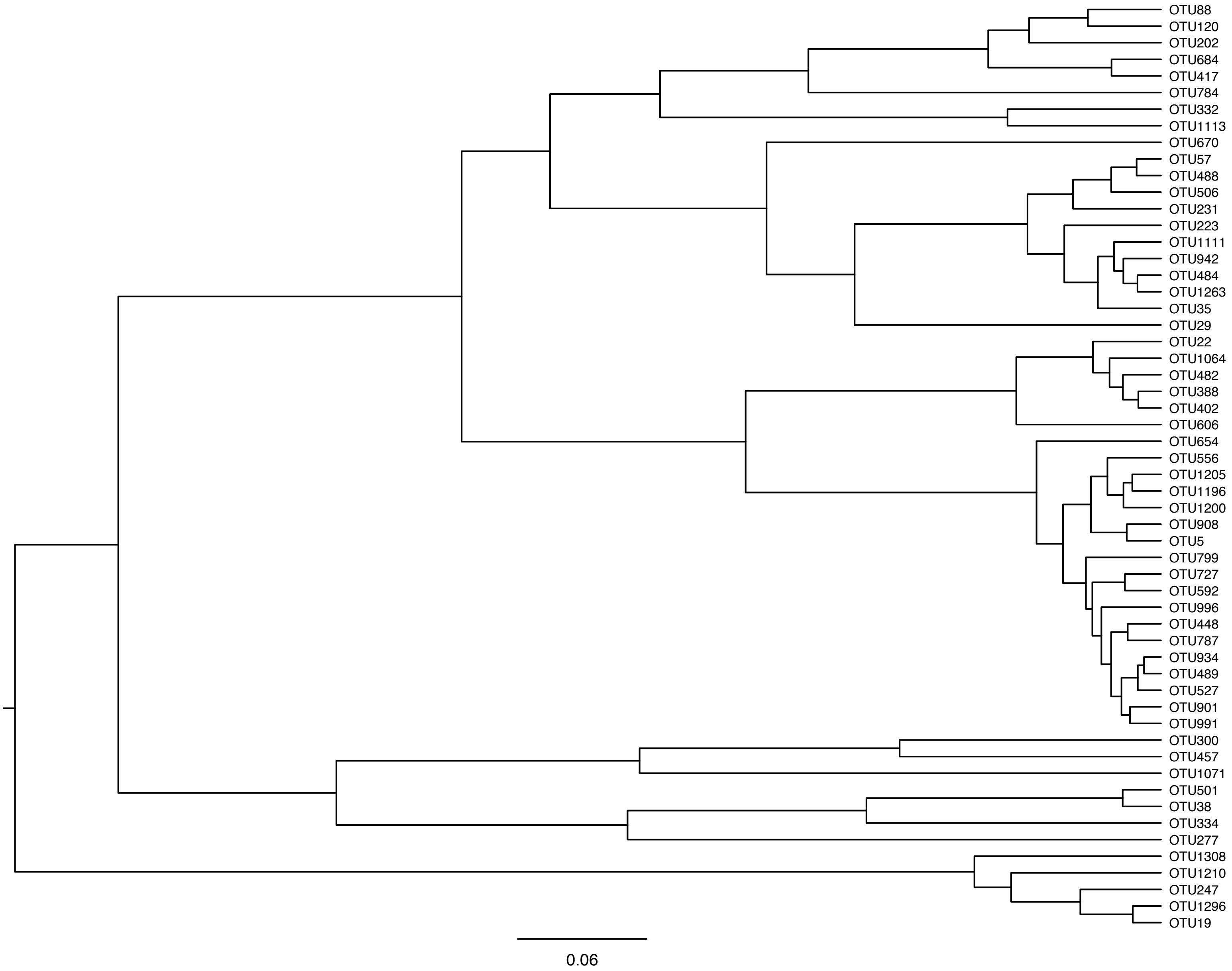

Supplementary Figure 21

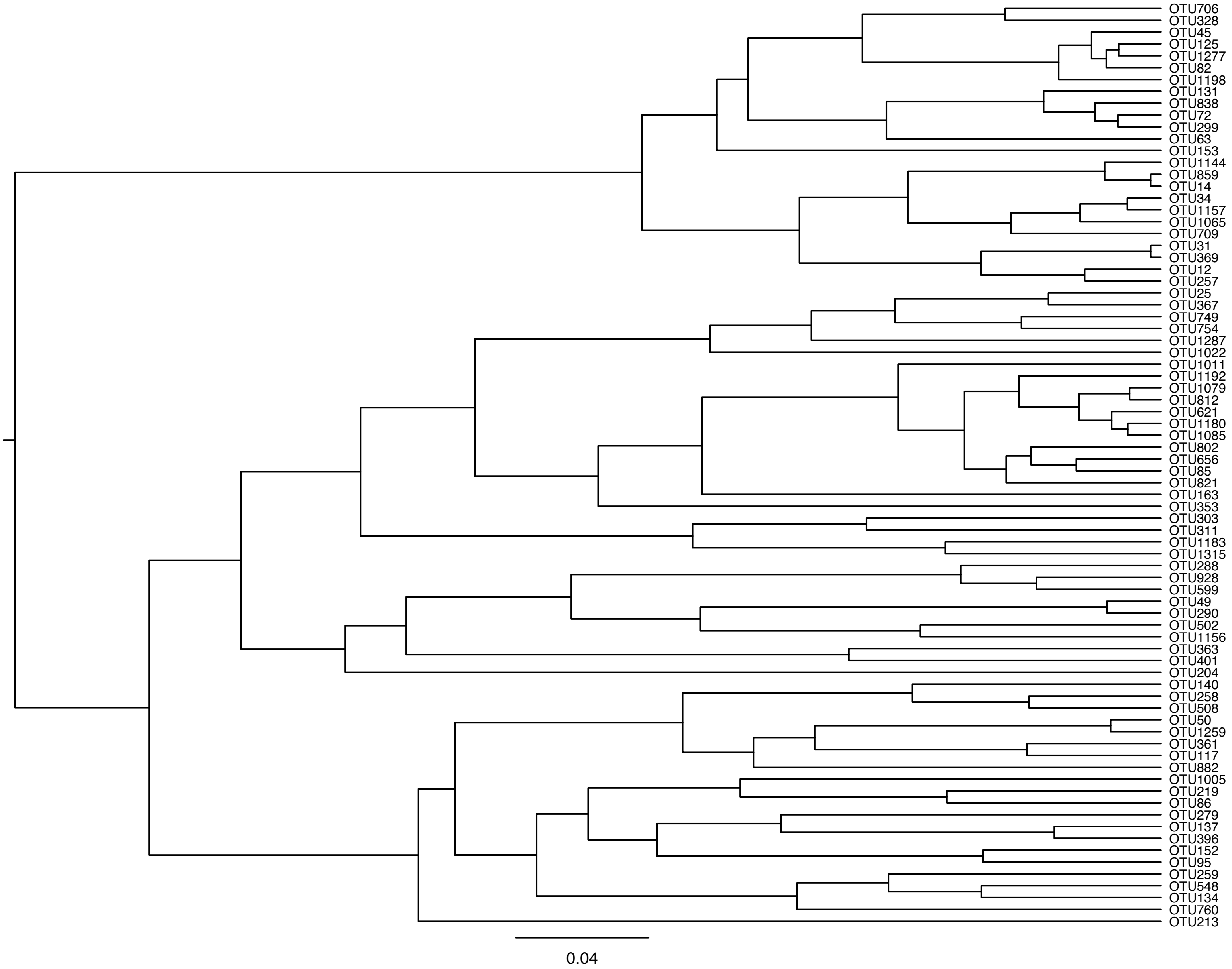

Supplementary Figure 22

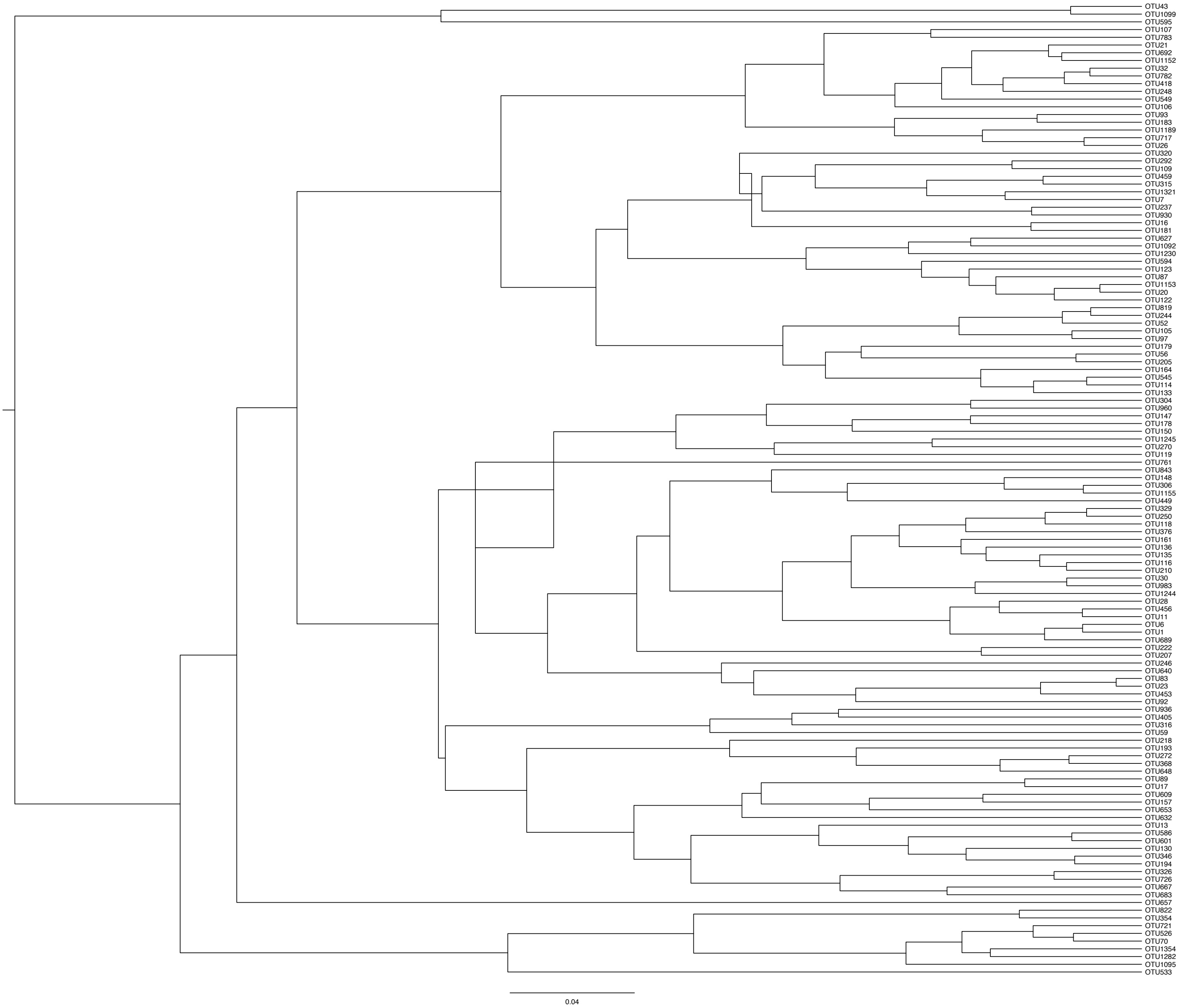

Supplementary Figure 23

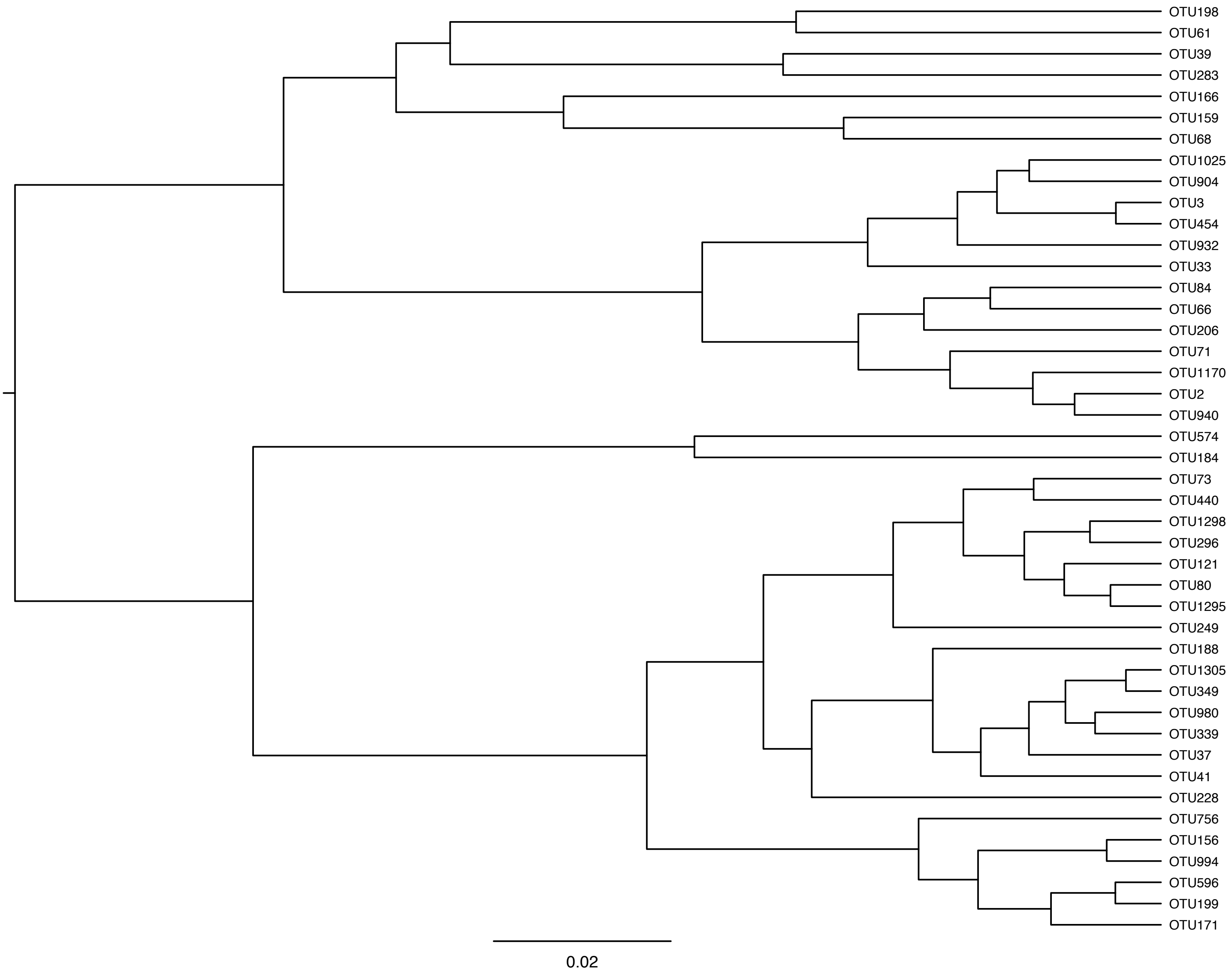

Supplementary Figure 24

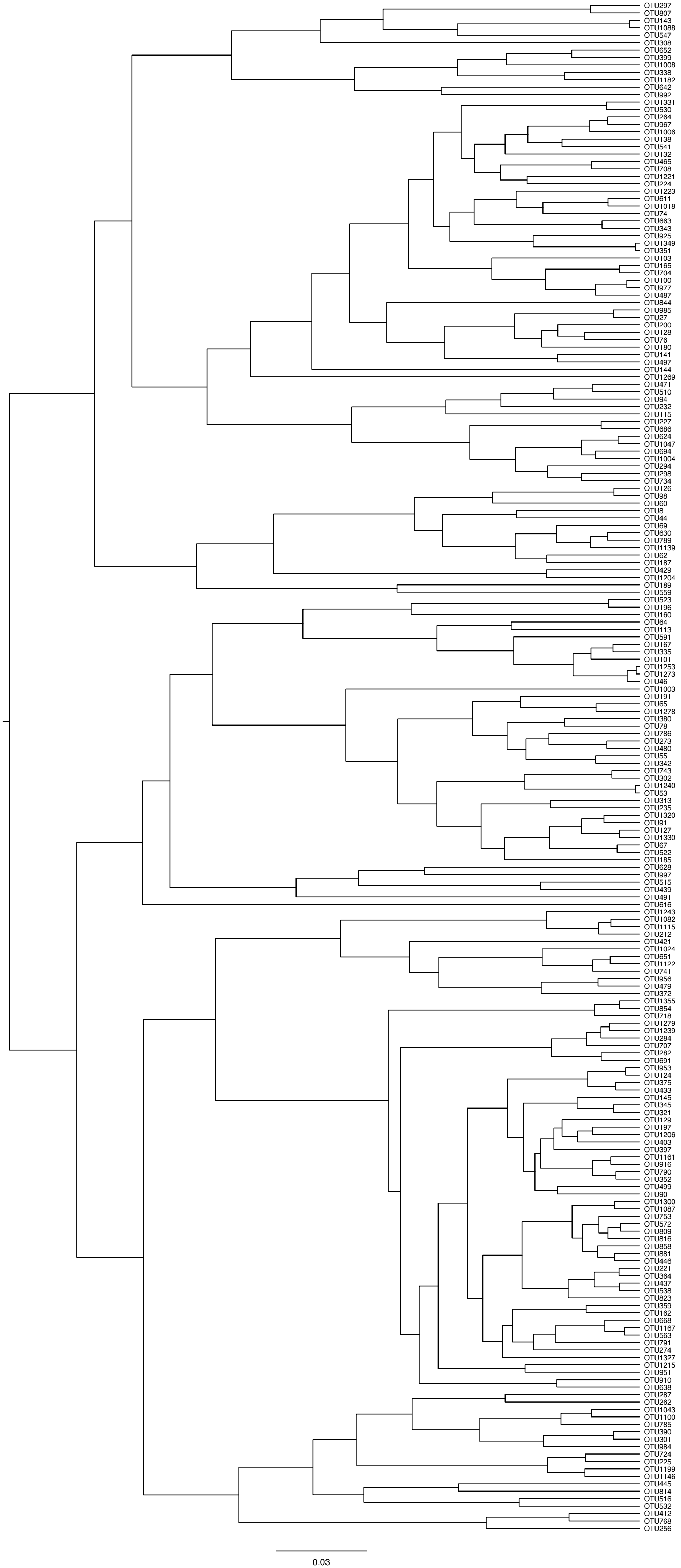

Supplementary Figure 25

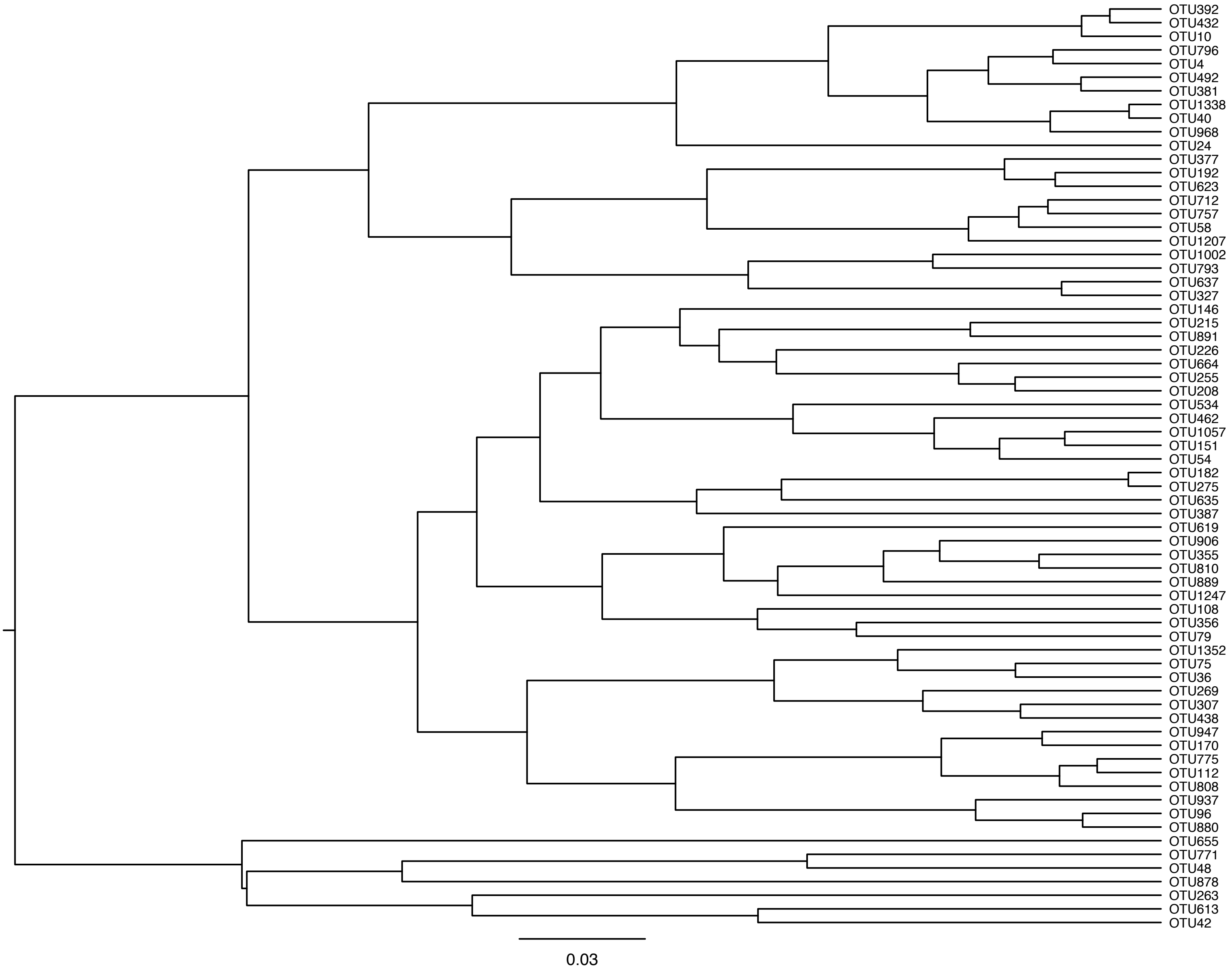

Supplementary Figure 26

Supplementary Figures

Supplementary Figure 1 Neighbour Joining tree of all ZOTUs.

Supplementary Figure 2 Neighbour Joining trees for Acarii, including the ZOTUs of this study and all available 18S sequences from GenBank.

Supplementary Figure 3 Neighbour Joining trees for Acoela, including the ZOTUs of this study and all available 18S sequences from GenBank.

Supplementary Figure 4 Neighbour Joining trees for Annelida, including the ZOTUs of this study and all available 18S sequences from GenBank.

Supplementary Figure 5 Neighbour Joining trees for Ascidiacea, including the ZOTUs of this study and all available 18S sequences from GenBank.

Supplementary Figure 6 Neighbour Joining trees for Cnidaria, including the ZOTUs of this study and all available 18S sequences from GenBank.

Supplementary Figure 7 Neighbour Joining trees for Crustacea Copepoda, including the ZOTUs of this study and all available 18S sequences from GenBank.

Supplementary Figure 8 Neighbour Joining trees for Gastrotricha, including the ZOTUs of this study and all available 18S sequences from GenBank.

Supplementary Figure 9 Neighbour Joining trees for Gnathostomulida, including the ZOTUs of this study and all available 18S sequences from GenBank.

Supplementary Figure 10 Neighbour Joining trees for Kinorhyncha, including the ZOTUs of this study and all available 18S sequences from GenBank.

Supplementary Figure 11 Neighbour Joining trees for Mollusca, including the ZOTUs of this study and all available 18S sequences from GenBank.

Supplementary Figure 12 Neighbour Joining trees for Mysida, including the ZOTUs of this study and all available 18S sequences from GenBank.

Supplementary Figure 13 Neighbour Joining trees for Nematoda, including the ZOTUs of this study and all available 18S sequences from GenBank.

Supplementary Figure 14 Neighbour Joining trees for Nemertea, including the ZOTUs of this study and all available 18S sequences from GenBank.

Supplementary Figure 15 Neighbour Joining trees for Crustacea Ostracoda, including the ZOTUs of this study and all available 18S sequences from GenBank.

Supplementary Figure 16 Neighbour Joining trees for Platyhelminthes, including the ZOTUs of this study and all available 18S sequences from GenBank.

Supplementary Figure 17 Neighbour Joining trees for Porifera, including the ZOTUs of this study and all available 18S sequences from GenBank.

Supplementary Figure 18 Neighbour Joining trees for Phoronida, including the ZOTUs of this study and all available 18S sequences from GenBank.

Supplementary Figure 19 Neighbour Joining trees for Rotifera, including the ZOTUs of this study and all available 18S sequences from GenBank.

Supplementary Figure 20 Neighbour Joining trees for Tardigrada, including the ZOTUs of this study and all available 18S sequences from GenBank.

Supplementary Figure 21 BEAST tree of zOTUs from Acoela.

Supplementary Figure 22 BEAST tree of zOTUs from Annelida.

Supplementary Figure 23 BEAST tree of zOTUs from Crustacea Copepoda.

Supplementary Figure 24 BEAST tree of zOTUs from Gastrotricha.

Supplementary Figure 25 BEAST tree of zOTUs from Nematoda.

Supplementary Figure 26 BEAST tree of zOTUs from Platyhelminthes.

Supplementary Tables

Supplementary Table 1 Summary of the BLAST iterative approach for the assignment of MOTUs to metazoan meiofaunal groups. Blast = number of metazoan MOTUs unequivocally identified using Blast. NJ = number of MOTUs that we could not identify using Blast, but we could place phylogenetically using Neighbour Joining trees on all available GenBank sequences (Supplementary Figures 1-20). GB = number of incorrectly identified MOTUs by Blast, detected using Neighbour Joining trees on all available GenBank sequences. Analysis = number of MOTUs included in the group-specific analyses, when excluding the non-meiofaunal representative of metazoans; in the last column, “NA” means that the group was indeed from meiofauna but was not included in the group-specific analyses, “-“ means that the group is not strictly meiofaunal.

	Blast	NJ	GB	Analysis
Acarii	5	0	0	NA
Acoela	54	2	1	55
Annelida	78	1	0	79
Ascidiacea	1	0	0	-
Cnidaria	8	0	0	-
Copepoda	69	57	0	126
Gastrotricha	38	6	0	44
Gnathostomulida	13	0	0	NA
Kinorhyncha	1	0	0	NA
Mollusca	6	0	0	NA
Mysida	1	0	0	-
Nematoda	185	21	0	206
Nemertea	14	0	0	NA
Ostracoda	9	7	0	NA
Platyhelminthes	66	0	0	66
Porifera	1	0	0	-
Phoronida	1	0	0	-
Rotifera	3	0	0	NA
Tardigrada	6	0	0	NA
Vertebrata	1	0	0	-

Supplementary Table 2 Analysis of Deviance Table (Type II tests) obtained from generalized linear models, including total number of MOTUs or MOTUs per main phyla as response variable, and depth and beach as explanatory variables. Abbrevations: LR Chisq = likelihood ratio chi-square values; df = degrees of freedom; P = chi-square goodness of fit.

		LR Chisq	df	P
Total	depth	25.419	2	<0.0001
	beach	31.36	10	0.0005
	number of reads	0.772	1	0.379
Acoela	depth	1.065	2	0.587
	beach	30.606	10	0.0007
	number of reads	5.379	1	0.02
Annelida	depth	22.214	2	<0.0001
	beach	35.525	10	0.0001
	number of reads	0.183	1	0.669
Copepoda	depth	14.014	2	0.0001
	beach	9.153	10	0.518
	number of reads	0.896	1	0.344
Gastrotricha	depth	10.621	2	0.005
	beach	9.094	10	0.523
	number of reads	0.106	1	0.745
Nematoda	depth	10.603	2	0.005
	beach	33.039	10	0.0002
	number of reads	1.395	1	0.238
Platyhelminthes	depth	17.925	2	0.0001
	beach	61.192	10	<0.0001
	number of reads	2.125	1	0.145

Supplementary Table 3 Analysis of Deviance Table (Type II tests) obtained from generalized linear models (GLMs) to test the effect of the number of tourists together with the potential confounding factors of granulometry and beach length on the richness of MOTUs for each meiofaunal group at the three water depth levels. Abbrevations: LR Chisq = likelihood ratio chi-square values; estimate = coefficient for the regression model; df, degrees of freedom; P = chi-square goodness of fit.

	Depth	Predictor	LR Chisq	estimate	df	P
Acoela	Swash	tourists	0.0001	0.003	1	0.990
		granulometry	NA	NA	NA	NA
		length	0.087	-0.077	1	0.769
	Shallow	tourists	5.918	-23.100	1	**0.015**
		granulometry	7.848	NA	4	0.097
		length	6.725	-27.265	1	**0.009**
	Deep	tourists	3.139	0.407	1	0.076
		granulometry	14.156	NA	4	0.007
		length	0.439	0.099	1	0.508
Annelida	Swash	tourists	0.055	-0.054	1	0.815
		granulometry	NA	NA	NA	NA
		length	1.856	0.271	1	0.173
	Shallow	tourists	0.157	-0.321	1	0.693
		granulometry	11.104	NA	4	**0.025**
		length	0.242	-0.382	1	0.623
	Deep	tourists	0.268	0.069	1	0.605
		granulometry	28.631	NA	4	**<0.0001**
		length	0.934	-0.127	1	0.334
Copepoda	Swash	tourists	4.603	-0.419	1	**0.031**
		granulometry	NA	NA	NA	NA
		length	1.868	0.205	1	0.172
	Shallow	tourists	0.0001	0.006	1	0.999
		granulometry	2.178	NA	4	0.703
		length	0.169	0.199	1	0.681
	Deep	tourists	0.660	0.072	1	0.417
		granulometry	38.325	NA	4	**<0.0001**
		length	1.190	0.090	1	0.275
Gastrotricha	Swash	tourists	0.076	-0.056	1	0.783
		granulometry	NA	NA	NA	NA
		length	0.002	-0.009	1	0.963

	Shallow	tourists	0.248	0.638	1	0.619
		granulometry	3.645	NA	4	0.456
		length	0.000	-0.005	1	0.997
	Deep	tourists	1.091	0.145	1	0.296
		granulometry	1.453	NA	4	0.835
		length	4.049	0.273	1	**0.044**
Nematoda	Swash	tourists	1.025	-0.167	1	0.312
		granulometry	NA	NA	NA	NA
		length	2.714	-0.288	1	0.100
	Shallow	tourists	0.517	0.664	1	0.472
		granulometry	92.591	NA	4	**<0.0001**
		length	0.615	0.786	1	0.424
	Deep	tourists	0.185	-0.038	1	0.667
		granulometry	71.677	NA	4	**<0.0001**
		length	3.953	-0.156	1	**0.047**
Platyhelminthes	Swash	tourists	0.006	0.017	1	0.938
		granulometry	NA	NA	NA	NA
		length	0.536	0.154	1	0.464
	Shallow	tourists	0.414	0.584	1	0.520
		granulometry	3.973	NA	4	0.410
		length	0.109	0.292	1	0.741
	Deep	tourists	8.540	0.390	1	**0.003**
		granulometry	15.123	NA	4	**0.004**
		length	0.238	0.078	1	0.626

Supplementary Table 4 Analysis of Deviance Table (Type II tests) obtained from generalized linear models (GLMs) to test the effect of the number of tourists together with the potential confounding factor of granulometry and beach length on the richness of morphological species for each meiofaunal group at the three depth levels. Abbrevations: LR Chisq = likelih0od ratio chi-square values; df, degrees of freedom; estimate, coeficient for the regression model; P = chi-square goodness of fit.

			LR Chisq	estimate	df	P
Total species	Swash	tourists	2.611	0.663	1	0.1062
		granulometry	NA	NA	NA	NA
		length	1.685	0.670	1	0.1943
	Shallow	tourists	-	0.996	-	0.0622
		granulometry	1.838	NA	2	0.1392
		length	-	1.357	-	0.2996
	Deep	tourists	1.91	0.094	1	0.1672
		granulometry	343.5	NA	2	**<0.0001**
		length	147.18	0.925	1	**<0.0001**
Acoela	Swash	tourists	0.893	0.767	1	0.3447
		granulometry	NA	NA	NA	NA
		length	0.058	0.290	1	0.8090
	Shallow	tourists	-	-0.255	-	0.8950
		granulometry	0.006	NA	2	0.9967
		length	-	0.026	-	0.9910
	Deep	tourists	87.401	2.818	1	**<0.0001**
		granulometry	85.87	NA	2	**<0.0001**
		length	144.725	4.373	1	**<0.0001**
Annelida	Swash	tourists	0.000	25.184	1	**<0.0001**
		granulometry	NA	NA	NA	NA
		length	0.000	30.966	1	**<0.0001**
	Shallow	tourists	-	-0.510	-	0.7900
		granulometry	1.839	NA	2	0.3987
		length	-	0..051	-	0.9820
	Deep	tourists	10.225	2.092	1	0.001
		granulometry	32.137	NA	2	**<0.0001**
		length	15.751	3.271	1	**<0.0001**
Gastrotricha	Swash	tourists	2.181	0.529	1	0.1397
		granulometry	NA	NA	NA	NA
		length	0.323	0.285	1	0.5698

	Shallow	tourists	-	44.913	-	0.9990
		granulometry	6.797	NA	2	**0.0330**
		length	-	52.994	-	0.9990
	Deep	tourists	1.858	-0.831	1	0.1728
		granulometry	14.669	NA	2	**0.0006**
		length	3.883	0.860	1	0.0488
Platyhelminthes	Swash	tourists	0.066	0.767	1	0.7977
		granulometry	NA	NA	NA	NA
		length	0.103	0.975	1	0.7483
	Shallow	tourists	-	-0.206	-	0.9130
		granulometry	4.229	NA	2	0.1207
		length	-	0.021	-	0.9926
	Deep	tourists	0.216	-0-106	1	0.6421
		granulometry	17.967	NA	2	**0.0001**
		length	4.268	0.551	1	**0.0388**

Supplementary Table 5 Summary of the R^2 and P values from the permutational multivariate analyses of variance (PERMANOVA) obtained with adonis using Jaccard Dissimilarity index calculated for MOTUs assemblages response variable, and number of tourists, granulometry, depth, and beach as explanatory variables.

	Tourists		Granulometry		Depth		Beach		
	R^2	P	R^2	P	R^2	P	R^2	P	residual R^2
Acoela	0.083	**0.0171**	0.293	0.0993	0.060	0.769	0.378	**0.0281**	0.187
Annelida	0.041	0.264	0.250	0.1254	0.110	**0.0053**	0.384	0.0646	0.216
Copepoda	0.059	**0.0056**	0.276	**0.0016**	0.100	**0.0034**	0.409	**0.0016**	0.155
Gastrotricha	0.045	0.115	0.272	**0.0204**	0.111	**0.0101**	0.381	**0.0362**	0.190
Nematoda	0.063	**0.0041**	0.260	**0.0184**	0.093	**0.0256**	0.371	0.0578	0.213
Platyhelminthes	0.045	0.174	0.245	0.1429	0.089	0.1035	0.409	**0.0320**	0.211

Supplementary Table 6 Summary of the R^2 and P values from the permutational multivariate analyses of variance obtained with adonis using Jaccard Dissimilarity index calculated for MOTUs assemblages at each depth level as response variable and number of tourists and granulometry as explanatory variables.

		Tourists		Granulometry		
		R^2	P	R^2	P	residual R^2
Swash	Acoela	0.149	0.617	NA	NA	0.851
	Annelida	0.182	0.336	NA	NA	0.818
	Copepoda	0.216	0.325	NA	NA	0.784
	Gastrotricha	0.125	0.791	NA	NA	0.875
	Nematoda	0.221	0.051	NA	NA	0.779
	Platyhelminthes	0.182	0.335	NA	NA	0.818
Shallow	Acoela	0.120	0.773	0.710	0.440	0.170
	Annelida	0.154	0.660	0.667	0.613	0.179
	Copepoda	0.172	0.291	0.681	0.258	0.148
	Gastrotricha	0.195	0.035	0.732	0.061	0.072
	Nematoda		0.296	0.666	0.320	0.152
	Platyhelminthes	0.150	0.594	0.685	0.453	0.165
Deep	Acoela	0.113	0.187	0.414	0.279	0.463
	Annelida	0.096	0.375	0.451	0.091	0.453
	Copepoda	0.107	0.326	0.404	0.427	0.489
	Gastrotricha	0.111	0.325	0.340	0.447	0.489
	Nematoda	0.116	0.096	0.408	0.241	0.476
	Platyhelminthes	0.084	0.715	0.420	0.317	0.497

Supplementary Table 7 Results of the Analysis of Deviance Table (Type II tests), obtained from generalized linear models, using phylogenetic diversity and phylogenetic sorting of each phylum as response variable, and depth, beach, and total number of MOTUs as explanatory variables. Abbrevations: LR-Chisq = likelihood ratio chi-square values; df, degrees of freedom; P = chi-square goodness of fit.

		Phylogenetic diversity			Phylogenetic sorting		
		LR Chisq	df	P	LR Chisq	df	P
Acoela	depth	3.118	2	0.2103	0.798	2	0.6709
	tourists	0.055	1	0.8152	0.967	1	0.3255
	granulometry	6.230	6	0.3979	4.411	6	0.6212
	length	0.009	1	0.9243	0.136	1	0.7123
	MOTUs	36.816	1	**<0.0001**	0.032	1	0.8591
	depth*tourists	1.904	2	0.3860	2.709	2	0.2580
Annelida	depth	6.360	2	**0.0416**	1.841	2	0.3982
	tourists	0.040	1	0.8414	0.030	1	0.8635
	granulometry	36.158	6	**<0.0001**	18.433	6	0.2707
	length	0.790	1	0.3741	1.213	1	**0.0052**
	MOTUs	4.189	1	**0.0407**	5.524	1	**0.0188**
	depth*tourists	3.881	2	0.1437	3.260	2	0.1959
Copepoda	depth	1.379	2	0.5019	1.876	2	0.3915
	tourists	1.936	1	0.1641	0.064	1	0.7999
	granulometry	4.100	6	0.8784	6.401	6	0.3799
	length	0.023	1	0.6631	0.737	1	0.3907
	MOTUs	109.086	1	**<0.0001**	0.228	1	0.6334
	depth*tourists	0.149	2	0.9282	2.258	2	0.3234
Gastrotricha	depth	0.421	2	0.8103	0.503	2	0.7775
	tourists	0.081	1	0.7761	0.018	1	0.8948
	granulometry	1.351	6	0.9688	3.666	6	0.7218
	length	5.377	1	**0.0204**	2.205	1	0.1376
	MOTUs	21.715	1	**<0.0001**	0.005	1	0.9417
	depth*tourists	3.976	2	0.1370	3.951	2	0.1387
Nematoda	depth	1.881	2	0.3905	1.634	2	0.4417
	tourists	2.651	1	0.1035	0.143	1	0.7050
	granulometry	19.865	6	**0.0029**	3.098	6	0.7965
	length	2.864	1	0.0889	0.078	1	0.7804
	MOTUs	298.531	1	**<0.0001**	0.000	1	0.9887
	depth*tourists	0.062	2	0.9697	1.587	2	0.4523

Platyhelminthes	depth	3.988	2	0.1362	0.480	2	0.7867
	tourists	0.029	1	0.8648	0.044	1	0.8341
	granulometry	10.876	6	0.0923	11.476	6	0.2247
	length	0.776	1	0.3785	1.474	1	0.0747
	MOTUs	50.780	1	**<0.0001**	2.989	1	0.0838
	depth*tourists	4.458	2	0.1066	8.818	2	**0.0121**
