# Supplementary material for: Human access impacts biodiversity of microscopic animals in sandy beaches
Source: Commun Biol. 2020 Apr 20;3:175. doi: 10.1038/s42003-020-0912-6 (PMC7170908; doi:10.1038/s42003-020-0912-6)
Supplement: Supplementary file 2 — Reporting Summary [file 42003_2020_912_MOESM2_ESM.pdf]

nature research

Corresponding author(s): Diego Fontaneto

Last updated by author(s): Mar 3, 2020

# Reporting Summary

Nature Research wishes to improve the reproducibility of the work that we publish. This form provides structure for consistency and transparency in reporting. For further information on Nature Research policies, see Authors & Referees and the Editorial Policy Checklist.

## Statistics

For all statistical analyses, confirm that the following items are present in the figure legend, table legend, main text, or Methods section.

| n/a | Confirmed | |
|---|---|---|
| ☐ | ☒ | The exact sample size ($n$) for each experimental group/condition, given as a discrete number and unit of measurement |
| ☐ | ☒ | A statement on whether measurements were taken from distinct samples or whether the same sample was measured repeatedly |
| ☐ | ☒ | The statistical test(s) used AND whether they are one- or two-sided<br>*Only common tests should be described solely by name; describe more complex techniques in the Methods section.* |
| ☐ | ☒ | A description of all covariates tested |
| ☐ | ☒ | A description of any assumptions or corrections, such as tests of normality and adjustment for multiple comparisons |
| ☐ | ☒ | A full description of the statistical parameters including central tendency (e.g. means) or other basic estimates (e.g. regression coefficient) AND variation (e.g. standard deviation) or associated estimates of uncertainty (e.g. confidence intervals) |
| ☐ | ☒ | For null hypothesis testing, the test statistic (e.g. $F$, $t$, $r$) with confidence intervals, effect sizes, degrees of freedom and $P$ value noted<br>*Give P values as exact values whenever suitable.* |
| ☒ | ☐ | For Bayesian analysis, information on the choice of priors and Markov chain Monte Carlo settings |
| ☐ | ☒ | For hierarchical and complex designs, identification of the appropriate level for tests and full reporting of outcomes |
| ☐ | ☒ | Estimates of effect sizes (e.g. Cohen's $d$, Pearson's $r$), indicating how they were calculated |

*Our web collection on statistics for biologists contains articles on many of the points above.*

## Software and code

Policy information about availability of computer code

| | |
|---|---|
| Data collection | no software was used |
| Data analysis | R was used with several packages without any custom script |

For manuscripts utilizing custom algorithms or software that are central to the research but not yet described in published literature, software must be made available to editors/reviewers. We strongly encourage code deposition in a community repository (e.g. GitHub). See the Nature Research guidelines for submitting code & software for further information.

## Data

Policy information about availability of data

All manuscripts must include a data availability statement. This statement should provide the following information, where applicable:

- Accession codes, unique identifiers, or web links for publicly available datasets
- A list of figures that have associated raw data
- A description of any restrictions on data availability

*Provide your data availability statement here.*

# Field-specific reporting

Please select the one below that is the best fit for your research. If you are not sure, read the appropriate sections before making your selection.

☐ Life sciences ☐ Behavioural & social sciences ☒ Ecological, evolutionary & environmental sciences

For a reference copy of the document with all sections, see nature.com/documents/nr-reporting-summary-flat.pdf

# Ecological, evolutionary & environmental sciences study design

All studies must disclose on these points even when the disclosure is negative.

| | |
|---|---|
| Study description | meiofauna extracted from three depth levels nested within replicated beaches |
| Research sample | A group of 11 beaches |
| Sampling strategy | all beaches longer than 10m in the island were sampled |
| Data collection | sediment samples were collected and used for DNA extraction, identification of meiofauna from morphology., and analysis of sediment texture |
| Timing and spatial scale | within two weeks all beaches in the island were sampled, within a maximum geographic distance of 15.5km |
| Data exclusions | no data were excluded a priori |
| Reproducibility | as replicable as a field study can be: all beaches can be resampled |
| Randomization | not relevant because of spatially explicit design |
| Blinding | sample names were not identifiable to the beach by the people working on sediment analysis, on meiofauna identification, and on DNA amplification |

Did the study involve field work? ☒ Yes ☐ No

## Field work, collection and transport

| | |
|---|---|
| Field conditions | calm and usual weather conditions for the season |
| Location | all data is present in figure 1 and Supplementary Table 1 |
| Access and import/export | all permits were obtained from the National Park, with its authorities involved in all the steps of the project |
| Disturbance | only minimal disturbance by collecting jars of sediment |

# Reporting for specific materials, systems and methods

We require information from authors about some types of materials, experimental systems and methods used in many studies. Here, indicate whether each material, system or method listed is relevant to your study. If you are not sure if a list item applies to your research, read the appropriate section before selecting a response.

### Materials & experimental systems

| n/a | Involved in the study | |
|---|---|---|
| ☒ | ☐ | Antibodies |
| ☒ | ☐ | Eukaryotic cell lines |
| ☒ | ☐ | Palaeontology |
| ☐ | ☒ | Animals and other organisms |
| ☒ | ☐ | Human research participants |
| ☒ | ☐ | Clinical data |

### Methods

| n/a | Involved in the study | |
|---|---|---|
| ☒ | ☐ | ChIP-seq |
| ☒ | ☐ | Flow cytometry |
| ☒ | ☐ | MRI-based neuroimaging |

## Animals and other organisms

Policy information about studies involving animals; ARRIVE guidelines recommended for reporting animal research

| | |
|---|---|
| Laboratory animals | no laboratory animals |
| Wild animals | only meiofauna |
| Field-collected samples | extracted meiofauna was preserved i nethanol until DNA extraction |
| Ethics oversight | the authorities of the National Park guided us through the ethics |

Note that full information on the approval of the study protocol must also be provided in the manuscript.
