# Supplementary material for: Human access impacts biodiversity of microscopic animals in sandy beaches
Source: Commun Biol. 2020 Apr 20;3:175. doi: 10.1038/s42003-020-0912-6 (PMC7170908; doi:10.1038/s42003-020-0912-6)
Supplement: Supplementary file 6 — Description of Additional Supplementary Files [file 42003_2020_912_MOESM6_ESM.pdf]

Supplementary Data

Supplementary Data 1 Information on each sample station, with coordinates in WGS84 reference system, water depth level, protection level, maximum number of tourists per day every 10 square meters, number of total high quality reads, total MOTUs, and total metazoan MOTUs per sample, mean grain size, sorting coefficient, kurtosis, skewness, and granulometry group. The number of tourists in restricted beaches was scored as inapplicable, “NA”.

Supplementary Data 2 List of the morphological species, divided by main taxonomic group, and their distribution in each sample in each beach.
